# Hierarchical clustering for disentangling population vulnerability to asthma

**DOI:** 10.1016/j.envadv.2026.100735

**Published:** 2026-07-23

**Authors:** Xiang Gao, Parvathinathan Venkitasubramaniam, Hyunok Choi

**Affiliations:** Lehigh University, Bethlehem, PA, USA

**Keywords:** Structural clustering, Population vulnerability, Air pollution, Asthma hospitalization, Environmental health

## Abstract

Asthma is influenced by air pollution and weather conditions, yet asthma-related responses to these exposures may vary across communities due to heterogeneity in population vulnerability—defined here as a context represented by socioeconomic, demographic, and landscape conditions. We propose a clustering framework to characterize region-level population vulnerability prior to exposure–asthma modeling. Using statewide longitudinal data from Pennsylvania (2011–2019), socioeconomic, demographic, and landscape factors are aggregated at the region level and clustered through a hierarchical algorithm to define distinct population types. The algorithm combines outcome-informed feature weighting with agglomerative hierarchical clustering based on Euclidean distance and Ward linkage. These groups are subsequently incorporated into stratified modeling of age-adjusted asthma hospitalization rates in relation to pollutant and weather conditions. Compared with statewide pooled modeling, the group-specific approach improves exposure–asthma characterization and predictive performance, with an absolute overall increase in R^2^ of 0.064. Five coherent population types are identified, spanning upland–plateau, lowland–valley, affluent suburban, peri-urban, and urban-core settings and reflecting multidimensional vulnerability profiles. These findings suggest that population clustering provides a principled approach to vulnerability-based stratification, supporting a more differentiated assessment of asthma burden. The proposed methodology may be transferable to broader geographic settings and related respiratory health applications.

## 1. Introduction

Asthma is a prevalent chronic respiratory condition worldwide, affecting hundreds of millions of individuals (Goldin and Cataletto, 2026). Severe exacerbations can lead to emergency department visits, hospitalization, and even mortality, and they impose considerable healthcare and economic burdens (Qin et al., 2021; Lassenius et al., 2025). As reported in numerous epidemiological studies, asthma exacerbations have been linked to a range of ambient air pollutants, including particulate matter (PM_2.5_), sulfur dioxide (SO_2_), and nitrogen dioxide (NO_2_), among others (Meek et al., 2023; Zheng et al., 2021; Lai et al., 2018). In addition, changes in weather conditions—such as temperature and humidity—have also been shown to influence exacerbation risk (Romaszko-Wojtowicz et al., 2020). Importantly, the associations between these exposures and asthma outcomes may vary across communities, with multiple studies suggesting potential relevance of broader structural conditions. For example, Guo et al. (2022) showed that asthma prevalence differed across urban–rural classifications, reflecting geographic dimensions of population differences. Similarly, Cardet et al. (2018) examined asthma exacerbations and treatment failure in relation to household income levels. Landscape-focused studies have also connected residential altitude and greenness to asthma hospitalization and childhood asthma outcomes (Kiechl-Kohlendorfer et al., 2007; Hartley et al., 2020). These studies indicate that exposure-related asthma responses may depend in part on underlying population vulnerability. However, they generally focus on specific dimensions rather than integrating multiple structural factors into a broader context. In this work, we define population vulnerability as a region-level structural context represented by socioeconomic, demographic, and landscape conditions. To better capture heterogeneous exposure-related asthma responses across communities, vulnerability is operationalized through multidimensional structural profiles.

A related line of research has sought to more systematically quantify population vulnerability through composite indices for asthma analysis. For example, Molina et al. (2020) employed the Area Deprivation Index (ADI) as a proxy for socioeconomic vulnerability to assess its association with asthma severity. In addition, Kannoth et al. (2023) specifically developed a Neighborhood Environmental Vulnerability Index (NEVI) for asthma morbidity, and leveraged the index to capture variation in pediatric asthma emergency department (ED) visits. These methods provide practical and interpretable summaries that can support area ranking, identification of priority locations, and public health surveillance. However, due to the reduction of multidimensional conditions to an overall score, they may be less able to distinguish communities with similar overall vulnerability levels but different structural compositions. For example, one community may have elevated vulnerability primarily because of lower income, whereas another may receive a similar score because of higher population density and older age composition. Although similar on an index scale, such communities may represent different vulnerability profiles for exposure–asthma relationships. In this sense, clustering provides a complementary approach: rather than ranking communities along a scalar index, it groups communities according to multidimensional structural contexts and helps identify qualitatively different vulnerability profiles.

Building on this distinction, we propose a framework to identify distinct population types using structural features. In this framework, vulnerability is characterized through data-driven clustering, where populations are partitioned into structurally coherent groups. Within each group, residents share similar structural attributes and are hypothesized to experience comparable vulnerability patterns, whereas different groups are expected to represent distinct vulnerability profiles. This formulation uses vulnerability as a stratification basis for examining whether exposure–asthma relationships differ across structurally defined population groups. At one level, it preserves nonlinearity among variables related to socioeconomic position, geographic location, and landscape features. At another, it yields interpretable population types for comparison (e.g., urban low-income vs suburban high-income populations), enabling differences in vulnerability to be interrogated across groups. After the population groups are defined, pollutant and meteorological factors are examined in relation to asthma hospitalization outcomes through downstream modeling. In this stage, statewide pooled and group-specific models are compared to assess whether stratification yields clearer exposure–asthma relationships. The implementation builds on population-level data from Pennsylvania spanning 2011–2019. Structural characteristics underpin the clustering stage, where hierarchical clustering is adopted to partition populations. Hierarchical clustering was selected because it can represent nested structures, does not assume spherical clusters, and produces deterministic partitions. These properties support the analysis of population types across aggregation levels without requiring separate solutions for each cluster number. By embedding vulnerability stratification into the analytical design, the framework is intended to reduce the risk that incomplete structural representation may oversimplify exposure patterns or allow dominant structural gradients to obscure exposure–health relationships in population-level analysis. Against this background, this study aims to identify structurally coherent population groups across Pennsylvania and assess whether vulnerability-based stratification improves the modeling of exposure–asthma hospitalization associations compared with pooled statewide analysis.

The remainder of this paper is organized as follows. Section 2 describes the data sources, preprocessing procedures, and the proposed hierarchical clustering framework with downstream validation. Section 3 presents the clustering solution, structural feature profiles of the identified groups, and downstream modeling results and analysis. Section 4 evaluates the robustness of the clustering framework under multiple perturbation settings. Section 5 interprets the main findings and discusses their implications. Finally, Section 6 summarizes the study and its contributions.

## 2. Methods

As part of the proposed methodology, the clustering analysis aims to identify spatial regions with similar socioeconomic and geographic characteristics in order to operationalize population vulnerability. Pennsylvania is selected as the study area because it exhibits a high prevalence of asthma and has been reported to rank among the highest in the United States in excess mortality attributable to air pollution (Pennsylvania Department of Health, 2025; McKeon et al., 2022). Because structural features may not contribute equally to vulnerability, feature weights are incorporated in the clustering step to emphasize asthma-relevant structural dimensions. The weights are derived from marginal correlations between each structural feature and the asthma outcome. This weighting strategy reduces reliance on equal feature contributions and helps orient clustering toward asthma-relevant vulnerability profiles. Both weighting and modeling datasets are defined through a one-time split. For each region, one annual observation is randomly selected from each quarter across the 2011–2019 period, yielding four observations per region for the weighting subset. These observations are used only to estimate the marginal correlations and are excluded from the main analysis dataset. Resulting weights guide the clustering process for all regions, while downstream exposure–asthma models are fitted and evaluated only on the remaining observations. This design helps mitigate information leakage and circular reasoning. To evaluate the influence and stability of the weighting procedure, an unweighted hierarchical clustering analysis and a 100-run weight stability analysis are conducted as sensitivity analyses. Overall, these analyses indicate that the main clustering structure is reasonably stable, with detailed results reported in Section 4.

### 2.1. Data collection

To characterize population vulnerability shaped by multiple structural factors, we constructed an integrated dataset combining socioeconomic surveys, topographic raster data, land cover products, and hospital admission records. These sources provide the inputs for clustering analysis and support a transparent and reproducible analytical framework.

#### 2.1.1. Socioeconomic survey

The American Community Survey (ACS) is used as the primary data source to represent the socioeconomic and demographic characteristics of spatial units in the clustering analysis (U.S. Census Bureau, 2026). The ACS provides annual information across multiple population and socioeconomic dimensions. Consistent with the scope of this study, we extract 5-year estimates at the ZIP Code Tabulation Areas (ZCTA) level for the period from 2011 to 2019. To capture factors relevant to asthma, a total of 24 variables are selected and grouped into three categories, including total population, population counts by household income brackets (e.g., <$10,000, $10,000–$14,999, etc.), and population counts by age groups (e.g., <5, 5–9, etc.). All ACS variables were filtered to Pennsylvania ZCTAs, providing complete spatial coverage for the study area.

#### 2.1.2. Topographic data

Topographic information is obtained from Digital Elevation Models (DEM), which digitally represent the Earth’s bare-ground surface (National Aeronautics and Space Administration, 2023a). For analytical purposes, DEM data serve to describe the topographic context of spatial units, including absolute elevation and local terrain variability. The topographic surface is therefore assumed to remain stable over the study time frame, and only the most recent version of the DEM product is utilized. The data are acquired using a consistent spatial processing approach, by defining a rectangular bounding area covering Pennsylvania. Given resource constraints, a spatial resolution of 1/3 arc-second (approximately 10 m) is applied to balance computational efficiency and topographic detail.

#### 2.1.3. Land cover

Land cover characteristics are derived from the Moderate Resolution Imaging Spectroradiometer (MODIS), which provides frequent satellite-based observations of the Earth’s surface (National Aeronautics and Space Administration, 2023b). In this study, the Normalized Difference Vegetation Index (NDVI) is adopted as the measure of regional greenness for the spatial units considered (National Aeronautics and Space Administration, 2023c). NDVI observations are collected at a spatial resolution of 250 m × 250 m, with a temporal frequency of approximately 1–2 days. The data are extracted over a rectangular bounding area encompassing Pennsylvania and span the period from 2011 to 2019.

#### 2.1.4. Hospitalization records

Hospitalization records are obtained from the Pennsylvania Health Care Cost Containment Council (PHC4), which provides statewide inpatient discharge records from hospitals and freestanding ambulatory surgery centers (Pennsylvania Health Care Cost Containment Council, 2026). Due to privacy and data security considerations, ZIP code units are used as the spatial basis for deriving population-level health statistics, and their correspondence with ZCTAs facilitates spatial alignment with other datasets. The records are collected quarterly from 2011 to 2019. In this study, inpatient asthma hospitalization is used as the primary health outcome, capturing more severe disease episodes and reducing potential bias from differences in care-seeking behavior. Asthma cases are identified using ICD-9 and ICD-10 diagnosis codes, ensuring reliable classification while minimizing contamination from non-specific respiratory conditions (Centers for Disease Control and Prevention, 2026). These outcomes are used to derive asthma hospitalization rates and to support outcome-informed feature weighting in the clustering analysis.

### 2.2. Data preparation

#### 2.2.1. Data cleaning

Prior to clustering analysis, filtering and cleaning operations are necessary to eliminate observations that are incomplete, out of scope, or potentially affected by measurement artifacts. Socioeconomic variables are derived from ACS 5-year estimates, which represent pooled data over the preceding five years and provide more statistically stable and reliable measurements than single-year estimates at small geographic scales like ZCTAs. Invalid or missing raster values are removed from topographic data following standard processing procedures. For land cover indicators, pixels flagged as snow or ice are excluded to prevent contamination by abnormal weather conditions. Hospitalization records are retained only for ZIP codes located within Pennsylvania, and asthma admissions are identified using standardized ICD-9 and ICD-10 diagnosis codes, ensuring that included cases are consistently defined and geographically restricted to the study area. Specifically, asthma admissions are defined using ICD-9-CM codes 493.00–493.99 for hospitalization records from 2011 through the third quarter of 2015, and ICD-10-CM codes J45.0–J45.998 for records from the fourth quarter of 2015 through 2019, corresponding to the transition in diagnostic coding systems.

#### 2.2.2. Spatial and temporal harmonization

The heterogeneity of raw data sources requires harmonization before clustering and downstream modeling. Therefore, all datasets were transformed into consistent spatial and temporal analytical units. The overall data preparation and analytical workflow is illustrated in Fig. 1.

Temporally, quarterly resolution was selected as the common analytical granularity because asthma hospitalization outcomes were available on a quarterly basis. Variables with seasonal variability, including land cover indicators, were aggregated to quarterly summaries, whereas socioeconomic survey data were retained at the annual level and topographic variables were treated as time-invariant. The final analytical panel contained 36 quarterly observations from 2011 to 2019.

Spatially, all datasets are aligned to aggregated ZCTA-based regions. This choice is constrained by the hospitalization records, for which ZIP code is the finest consistently available geographic identifier under privacy and confidentiality regulations. However, direct quarterly analysis at the ZCTA level would be affected by sparse asthma hospitalization counts, unstable ACS-derived estimates, and frequent zero values, especially in small-population areas. Such zero inflation can reduce the stability and interpretability of asthma burden estimation, structural feature construction, and downstream modeling. Geographic aggregation is therefore used to improve statistical reliability while preserving as much spatial resolution as possible (Washington State Department of Health, 2018; Werner and Strosnider, 2020).

Accordingly, ZCTAs are aggregated into larger regions using a data-driven regionalization framework that does not incorporate asthma hospitalization outcomes. The procedure follows the regionalization algorithm proposed by Spielman and Folch (2015), which was specifically designed to reduce uncertainty in American Community Survey estimates. By using an internal-variance-minimizing objective, the algorithm supports the construction of stable and internally homogeneous regional partitions, providing a suitable basis for spatial aggregation in the present analysis. Each resulting region is required to maintain at least 5000 residents in all study years, restrict merging to spatially adjacent ZCTAs, prioritize structural similarity, and minimize unnecessary aggregation to preserve geographic detail. Geographic adjacency is defined using rook contiguity, in which two ZCTAs are considered adjacent only if they share a boundary segment. After regionalization, 745 final regions are generated, and the resulting boundaries are fixed across all study years. Region population is generally stable over time, with only a small proportion of regions showing substantial first-to-last-year population change. The final region boundaries are shown in Figure S1 in the Supplementary Material. These regions serve as the fundamental spatial unit for all subsequent analyses: hospitalization counts and ACS population statistics are aggregated to the region level, while topographic and land cover variables are summarized using zonal statistics.

#### 2.2.3. Structural variable construction

Following the definition of analysis units, structural variables are constructed to represent key socioeconomic, demographic, and landscape characteristics for clustering. Socioeconomic and demographic information from the ACS consists of detailed compositional variables, including household income categories (e.g., < $10, 000, $10, 000–$14, 999, …, ≥ $200, 000) and age distributions (e.g., under 5, 5–9, 10–14, …, over 85 years). Factor analysis is used to examine loading patterns among ACS composition variables and thereby guide their consolidation into income and age groups. To preserve interpretability, the final variables are constructed from the original proportions rather than using factor scores as model inputs. A three-factor solution is retained for both income and age variables because it explains more than 95% of the total variance while maintaining clear loading patterns. The detailed loading patterns are reported in Table 1.

For household income, the patterns distinguish lower-income categories below $50,000, middle-income categories between $50,000 and $99,999, and higher-income categories at or above $100,000. As a result, three income groups are derived to represent low-, middle-, and high-income households. For age composition, the loading patterns are less discrete but still indicate four candidate groups: children aged 0–14 years, young adults aged 15–24 years, adults aged 25–64 years, and older adults aged 65 years and above. The final feature set retains the proportions of children aged 0–14 years and older adults aged 65 years and above, because they are commonly recognized as vulnerable populations in asthma-related research. The young-adult and adult indicators are not retained because they provide limited additional separation of population vulnerability.

Although the prior regionalization ensures a minimum population of 5000 residents, regions still differ in both population size and geographic area. To account for remaining scale variation, population-based measures are expressed as proportions rather than absolute counts. In addition, population density, defined as total population divided by region area, is included to capture differences in settlement intensity.

Pennsylvania encompasses diverse landform types, including plateaus, plains, lowlands, and ridge-and-valley systems (Pennsylvania Department of Conservation and Natural Resources, 2026). To represent geographic variation, topographic and land cover information are summarized at the regional level. Mean elevation, elevation standard deviation, and NDVI are derived to reflect terrain characteristics and regional greenness. In total, nine structural variables are included: three income proportions, two age-group proportions, mean elevation, elevation variability, NDVI, and population density. These variables constitute the complete input set for the clustering analysis.

#### 2.2.4. Outcome definition

Asthma hospitalization serves as the primary outcome. Using inpatient discharge records, the outcome is quantified as the age-adjusted asthma hospitalization rate, defined as:

(1) $$r_{i}^{\left(r,t\right)}=\frac{H_{i}^{\left(r,t\right)}}{N_{i}^{\left(r,t\right)}}$$

(2) $$R_{\text{adj}}^{\left(r,t\right)}=\sum_{i=1}^{n}r_{i}^{\left(r,t\right)}\;\left(\frac{p_{i}}{P}\right)$$

where $r_{i}^{\left(r,t\right)}$ denotes the age-specific asthma hospitalization rate for age group *i* in region *r* during time period *t*, $R_{adj}^{\left(r,t\right)}$ denotes the corresponding age-adjusted asthma hospitalization rate for the same region and period, $H_{i}^{\left(r,t\right)}$ denotes the count of asthma-related inpatient hospitalizations for age group *i*, $N_{i}^{\left(r,t\right)}$ denotes the total population of age group *i* in the same region and period, and $p_{i}/P$ denotes the proportion of age group *i* in the U.S. standard population.

The presence of varying age structures across regions in Pennsylvania necessitates age adjustment to remove bias in observed hospitalization rates attributable to differences in population age composition (Centers for Disease Control and Prevention, 2025). The age-adjusted asthma hospitalization rate, derived from inpatient records, provides a standardized population-level measure of asthma burden. This rate is calculated separately within the weighting subset and the modeling subset. In the weighting subset, it is used to derive feature weights based on variable–outcome associations. In the modeling subset, the rate serves as the target for evaluating exposure–asthma relationships with air pollution and meteorological conditions.

### 2.3. Clustering methodology

#### 2.3.1. Algorithm

The hierarchical clustering algorithm used in the study was originally formalized by Ward (1963). It aims to organize *M* observations in an *N*-dimensional feature space into a hierarchical structure of clusters. The algorithm follows an agglomerative procedure, in which each observation initially forms its own cluster. At each step, the two most similar clusters are merged based on a specified linkage criterion, and this process continues until a complete hierarchical structure of the data is formed. Hierarchical clustering can represent nested partition structures, does not require clusters to be spherical, and provides a deterministic procedure whose results do not depend on the choice of starting points (Manning et al., 2008). This property is particularly important in our setting, where population vulnerability arises from complex interactions of socioeconomic, demographic, and landscape factors that may not conform to simple cluster geometries. In the public health domain, hierarchical clustering has been applied in varying contexts, including deriving patient subgroups from social-determinants-of-health profiles (Zhang et al., 2024) and identifying spatial clusters based on environmental stressors across small-area units (Vandeninden et al., 2024).

As alternative methods, k-means clustering and spectral clustering are examined using the same standardization and weighting strategy. The resulting partitions exhibit broadly similar large-scale spatial configurations, and the k-means solution at *k* = 5 closely resembles the hierarchical solution, indicating that the overall structure is not algorithm-specific. Spectral clustering, meanwhile, introduces a sharper separation between plateau areas and mountainous zones by emphasizing terrain discontinuities.

Despite these structural similarities, differences emerge in the consistency of the internal validity diagnostics used to determine the number of clusters. Under both k-means and spectral clustering, multiple criteria suggest different optimal resolutions, leading to ambiguity in choosing *k*. By contrast, hierarchical clustering with Ward’s linkage demonstrates convergence across these criteria at *k* = 5, providing a clearer resolution choice. From a methodological standpoint, k-means requires separate optimization for each *k* and is sensitive to initialization (Lloyd, 1982), while spectral clustering relies on graph construction and parameter specification (Ng et al., 2001). Hierarchical clustering yields a nested structure that embeds solutions at different values of *k* as successive aggregation levels. Given this property of enabling consistent evaluation across multiple interpretability and validation criteria, hierarchical clustering is adopted to partition regions based on the selected structural features.

#### 2.3.2. Normalization and weighting

For each structural variable, values are averaged over the study period to obtain region-level representations. These representations are standardized using z-scores to remove differences in measurement scale. As discussed in Section 1, structural characteristics are associated with underlying population vulnerability but may contribute with varying strength. Accordingly, Pearson correlation coefficients between each variable and the age-adjusted asthma hospitalization rate—computed prior to temporal averaging—are used to rescale the standardized representations, aligning their salience with relative contributions to vulnerability characterization. To preserve independence between the weighting and downstream modeling stages, the correlations are estimated from a subset of the hospitalization data. For each region, four quarterly observations are randomly selected to compute variable–outcome correlations, and the remaining observations are reserved for downstream exposure–asthma modeling.

### 2.4. Downstream validation

This study aims to reveal heterogeneity in exposure–asthma relationships across populations. Beyond clustering spatial units to form groups with similar underlying vulnerability profiles, an essential step is to characterize exposure–asthma associations. Specifically, we model the asthma outcome as a function of pollutant and meteorological factors:

(3) Asthma outcome∼f(pollutants, meteorological factors)

Previous studies have reported complex and nonlinear associations between these two components, motivating the use of flexible modeling approaches beyond traditional linear methods. To capture potentially complex dependencies among variables, machine learning models are employed to estimate the function f(⋅), where the age-adjusted rate serves as the dependent variable and the pollutant and meteorological levels serve as the predictors. The model development is conducted as a downstream task based on the resulting region clusters, comparing a statewide model $f_{PA}(\cdot )$ with group-specific models $f_{c}(\cdot )$. Although machine learning models typically lack direct interpretability, the clustering solution can be assessed from a heterogeneity perspective by examining disparities in predictive performance between these modeling settings.

Following the proposed functional formulation, the downstream task is defined as a supervised regression problem. Pollutant concentrations are obtained from the Community Multiscale Air Quality (CMAQ) modeling system (U.S. Environmental Protection Agency, 2024), including CO, SO_2_, NO_2_, O_3_, PM_2.5_, and PM_10_, while meteorological variables are derived from the Gridded Surface Meteorological Dataset (gridMET) (REACCH, 2016), including precipitation, temperature, humidity, and wind speed. Both datasets are originally at high spatial and temporal resolution, enabling aggregation to quarterly, region-level summaries aligned with the target definition.

In the training phase, Random Forest regressors are employed to model the statewide and group-specific data under two parallel configurations. Both employ identical feature sets, hyperparameters, and random seeds, and all modeling and evaluation procedures are applied consistently across different clustering solutions. Data splitting is performed using a group-aware splitting strategy at the region level, such that all observations from the same region are assigned exclusively to either the training or testing set, thereby preventing potential spatial information leakage.

In the evaluation phase, the splitting procedure is repeated 100 times with different random seeds to reduce variability in predictive performance induced by particular train–test partitions. For each split, predictive performance is evaluated using the coefficient of determination (*R*^2^) and the root mean squared error (RMSE). The statewide model is trained on the full training dataset ${𝒟}_{\text{train}}$ and evaluated on the corresponding testing dataset ${𝒟}_{\text{test}}$. In contrast, group-specific models are trained using only the training subsets associated with each cluster, denoted as ${𝒟}_{\text{train}}^{1},{𝒟}_{\text{train}}^{2},\ldots ,{𝒟}_{\text{train}}^{K}$. During evaluation, predictions and ground truth values obtained from each group-specific model are pooled across clusters to construct a unified testing set,

(4) $${\hat{D}}_{\text{test}}=\overset{K}{\underset{c=1}{\cup }}\left({\hat{y}}_{c},y_{c}\right)$$

which enables direct comparison with the statewide model evaluated on *D*_test_. Because the statewide and group-specific models are built independently, differences in their predictive performance reflect the effectiveness of the clustering solution.

## 3. Results

### 3.1. Selection of the number of clusters

Unlike partition-based unsupervised methods, hierarchical clustering can produce a full nested structure without predefining the cluster number. Consequently, the final selection is primarily guided by semantic interpretability and domain knowledge, as also suggested by Jain et al. (1999). In addition, we employ multiple internal validity criteria to evaluate the quality of the resulting clustering solution, aiming to balance real-world relevance with mathematical validity. Given these considerations, a clustering resolution with five clusters is selected. This resolution yields groups that display broadly coherent spatial patterns with discernible structural boundaries, providing a meaningful partition for characterizing heterogeneity in population vulnerability. Detailed interpretations of resulting cluster patterns are presented in Section 3.2.

Internal validity criteria further support this selection. Specifically, the Silhouette score and explained variability are used to assess the effect of different numbers of clusters. The Silhouette score evaluates clustering quality by contrasting the average distance between a sample and other points within the same cluster, denoted by *a*, with the average distance between that sample and points in its closest neighboring cluster, denoted by *b*. It is defined as

(5) $$\text{Silhouette}=\frac{b-a}{max(a,b)}$$

where *a* quantifies intra-cluster dissimilarity and *b* quantifies dissimilarity to the nearest neighboring cluster (Rousseeuw, 1987). The explained variability metric corresponds to the proportion of total variance explained by the clustering solution and is calculated as

(6) $$\text{Explained Variability}=1-\frac{WSS}{TSS}=\frac{BSS}{TSS}$$

where WSS and TSS denote the within-cluster and total sums of squares, respectively. This quantity reflects the variance-decomposition principle underlying variance-based cluster validity indices such as the Calinski–Harabasz criterion (Caliński and Harabasz, 1974). As the number of clusters increases, explained variability typically rises monotonically, while the marginal gain diminishes with additional clusters. Accordingly, the incremental change in explained variability is used to assess the trade-off between model complexity and variance explained. We tested clustering resolutions with *k* ranging from 3 to 10. The corresponding Silhouette scores, explained variability values, and incremental gains are summarized in Table 2. As shown in Figure S2(a) in the Supplementary Material, the Silhouette score attains its maximum at *k* = 3 (0.310) and remains similar across *k* = 4 to *k* = 6 (0.226, 0.224, and 0.224, respectively). Although the three-cluster solution shows the strongest average separation, it explains only 0.418 of the total variability and yields a broad partition. Such a coarse resolution, which only distinguishes rural, intermediate, and urban regions, is insufficient for distinguishing more specific vulnerability profiles. The explained-variability results provide additional support for a finer but still parsimonious solution. At *k* = 5, the clustering solution explains 0.589 of the total variability, and the gain from *k* = 4 to *k* = 5 remains meaningful (0.076). Substantively, this step separates a heterogeneous urban group into two structurally distinct profiles: the highest-density central urban regions and less dense but still urbanized surrounding regions. Because these profiles differ in population density, greenness, and socioeconomic composition, separating them provides a more informative basis for vulnerability stratification. In contrast, Figure S2(b) shows a marked reduction in incremental explained variability at *k* = 6, where the additional gain decreases to 0.025. The corresponding partition mainly subdivides an intermediate group into a large subgroup and a small subgroup containing only 15 regions, which is difficult to characterize and provides limited support for subsequent cluster-specific stratified modeling. Taken together, these results indicate that *k* = 5 balances cluster separation, explained variability, structural differentiation, and interpretability. After selecting the five-cluster solution, its stability is further evaluated through sensitivity analyses, as reported in Section 4.

### 3.2. Clustering solution

The final clustering solution divides 745 regions across Pennsylvania into five groups, spanning from highly urbanized areas to diverse non-urban landscapes. Distinct socioeconomic, demographic, and landscape patterns emerge across these groups, as illustrated in Fig. 2. These patterns are summarized as follows:

- *Upland–Plateau* (298 regions): The largest group, primarily covering the Appalachian portions of Pennsylvania, with extensive spatial coverage across upland and plateau areas.
- *Lowland–Valley* (191 regions): Mainly distributed across the Pennsylvania Piedmont, with additional presence in parts of Greater Pittsburgh and scattered low-lying valley areas in the state.
- *Affluent Suburb* (120 regions): Concentrated in suburban areas of Greater Philadelphia and the northern portion of Greater Pittsburgh, together with individual suburban areas adjacent to other major cities.
- *Peri-Urban* (93 regions): Located in urban areas beyond the most densely populated districts, including outer urban areas of larger cities such as Philadelphia and Pittsburgh and the broader urban areas of smaller cities like Erie and Scranton.
- *Urban Core* (43 regions): Representing the highest-density regions, primarily located in the central districts of Pennsylvania’s largest cities.

### 3.3. Structural feature profiles

Table 3 summarizes the socioeconomic, demographic, and landscape characteristics of the five identified groups, while Fig. 3 illustrates the distributions of the nine structural features across groups.

Income composition provides a clear socioeconomic contrast across groups. The Affluent Suburb group has the highest median proportion of high-income households (0.455) and the lowest median proportion of low-income households (0.260). In contrast, the Urban Core group shows the opposite pattern, with the highest low-income proportion (0.609) and one of the lowest high-income proportions (0.146). The Peri-Urban and Upland–Plateau groups also have elevated low-income proportions (0.535 and 0.526, respectively), whereas the Lowland–Valley group occupies a more intermediate position across income categories. Age composition shows more moderate separation than income composition. The proportion of residents aged 65 years and above is highest in the Upland–Plateau group (0.190) and lowest in the Urban Core group (0.113), while the proportion of residents aged 0–14 years varies within a narrower range across groups (0.163–0.187). These patterns indicate that age-related features contribute to group differentiation but exhibit greater overlap than income composition.

Population density provides the strongest structural contrast. The Urban Core group has by far the highest median population density (6048.720), followed by the Peri-Urban group (1545.924). The remaining groups have substantially lower densities, ranging from 138.735 in the Upland–Plateau group to 679.065 in the Affluent Suburb group. This separation is also visible in Fig. 3, where the density distribution of the Urban Core group is clearly shifted above those of all other groups. Landscape features further differentiate the groups. The Upland–Plateau group has the highest median mean elevation (399.038) and elevation variability (67.069), whereas the Urban Core group has the lowest values for both measures (69.711 and 10.816, respectively). NDVI also decreases along the urbanization gradient, from higher median values in the Upland–Plateau, Lowland–Valley, and Affluent Suburb groups (0.611, 0.593, and 0.593, respectively) to lower values in the Peri-Urban (0.453) and Urban Core (0.292) groups. Overall, the five groups are most clearly differentiated by income composition, population density, elevation, and vegetation, while age-related features show more partial overlap.

### 3.4. Clustering description

The five-cluster solution identifies distinct population types across Pennsylvania, each reflecting a different combination of socioeconomic position, demographic composition, landscape context, and settlement form. The Upland–Plateau group reflects the concentration of these regions in the Appalachian mountain and plateau areas, consistent with their elevated and topographically variable terrain, and may represent a rural upland structural context. Lowland–Valley regions correspond to lower-elevation valley and lowland settings, including the Pennsylvania Piedmont and gently sloping areas near Greater Pittsburgh, suggesting a lower-density non-urban context. The Affluent Suburb cluster captures the comparatively advantaged socioeconomic profile of this group and its suburban position within the broader metropolitan spheres of major cities, which may reflect a more resource-advantaged suburban context. Peri-Urban regions occupy an intermediate urban form: in larger metropolitan areas, they often correspond to peripheral urban zones, whereas in mid-sized cities, they may encompass much of the urbanized area. This pattern may represent a transitional urban context. Urban Core areas represent the most intensively urbanized population type and are concentrated primarily in the central districts of Pennsylvania’s largest cities, especially Philadelphia and Pittsburgh, suggesting a dense urban-core context. Together, these five population types distinguish multiple structural contexts, including rural upland, lower-density valley, advantaged suburban, transitional urban, and dense urban-core settings. These distinctions provide the basis for evaluating whether exposure–asthma relationships differ across population vulnerability contexts in the subsequent modeling analysis.

### 3.5. Downstream validation

#### 3.5.1. Outcome characteristics

With five distinct groups established, we proceed to downstream modeling to further examine heterogeneity in exposure–health relationships. As described in Section 2.2.4, the health outcome is proxied by the age-adjusted asthma hospitalization rate. The empirical distribution of this rate, as illustrated in Fig. 4, provides background information for understanding subsequent performance comparisons. Across all observations, the statewide mean rate is 226 cases per 100,000 population. The distribution exhibits asymmetry toward higher values, reflected in greater dispersion above the median and the presence of several high-end outliers corresponding to quarters in which certain regions experienced markedly elevated hospitalization rates.

A closer inspection within each group reveals differences in central tendency and variability. The Urban Core exhibits the greatest overall hospitalization burden, with the highest mean rate of 493 per 100,000 and a maximum observed value of 1329 per 100,000. The Peri-Urban forms a second tier (346 per 100,000), positioned below the Urban Core yet clearly higher than the others. Among the remaining groups, the Upland–Plateau records a mean rate of 204 per 100,000, most closely mirroring the statewide distribution. It is followed by the Lowland–Valley (181 per 100,000) and the Affluent Suburb (165 per 100,000), which shows the lowest hospitalization levels.

#### 3.5.2. Predictive performance comparison

In this stage, we compare the predictive performance of the statewide and group-specific modeling strategies across 100 random splits. Both models are evaluated at the overall level using the full testing set and at the group level using the corresponding group-specific testing subsets (see Section 2.4 for details). The average performance and the comparisons are summarized in Table 4.

At the overall level, the group-specific modeling yields a clear improvement across the same 100 repeated train–test splits, with paired 95% confidence intervals for both *ΔR*^2^ and *Δ*RMSE excluding zero. Mean *R*^2^ increases from 0.437 (SD = 0.107) under the statewide model to 0.501 (SD = 0.091) under the group-specific models, corresponding to a mean paired difference of 0.064 (95% CI: 0.040, 0.088). Mean RMSE decreases from 101.587 (SD = 7.882) to 97.193 (SD = 8.143), with a mean paired difference of −4.395 (95% CI: −6.428, −2.362). These results indicate that the overall improvement under the group-specific framework is consistent across repeated splits rather than being driven by a small number of random partitions.

Across individual groups, performance changes vary in both direction and magnitude. The most striking gains occur in the Affluent Suburb group, where the statewide model yields strongly negative *R*^2^ values. Since this group includes 120 regions and also shows a substantial RMSE reduction, the negative *R*^2^ is unlikely to be explained primarily by limited sample size or reduced within-group variance alone. Rather, this pattern suggests poor calibration under the pooled model, indicating that relationships learned from the full statewide dataset do not transfer well to this structurally distinct subgroup. Group-specific modeling corrects this predictive misalignment, restoring *R*^2^ to near-zero positive values and reducing RMSE by approximately 28.97 units. The Lowland–Valley and Peri-Urban groups also exhibit noticeable gains under the group-specific strategy, with *R*^2^ increasing from negative or near-zero values to approximately 0.15, pointing to stronger predictive alignment under stratification. By comparison, the Urban Core group shows inconsistent changes between the two modeling strategies, with *R*^2^ increasing while RMSE deteriorates slightly. This divergence might be related to the high dispersion and the presence of more frequent and extreme outliers within the group, as the two metrics respond differently to large deviations. The Upland–Plateau group shows negligible differences between the two strategies, which may stem from the similarity of its outcome distribution to the statewide pattern.

It is worth noting that the *R*^2^ values within each group are lower than the overall *R*^2^. This pattern is expected, as *R*^2^ depends on the variance of the response within each evaluation subset; when calculated within smaller subsets, the total variance is reduced, which naturally leads to lower *R*^2^ values. Therefore, *R*^2^ is not directly comparable across groups, and cross-group performance comparisons are more appropriately interpreted using absolute error measures such as RMSE. In aggregate, these comparisons demonstrate that the group-specific modeling strategy delivers superior predictive performance relative to statewide pooled modeling. This improvement may reflect heterogeneous population vulnerability in exposure-related asthma responses. When distinct populations are modeled together, the statewide model may smooth over group-specific exposure–asthma patterns and produce a relationship that is less representative of selected groups. By estimating models within more coherent vulnerability profiles, the group-specific strategy reduces this predictive misalignment and better captures group-specific exposure–asthma patterns.

## 4. Sensitivity analysis

The sensitivity analyses evaluate whether the main clustering structure is preserved under alternative weighting specifications, repeated weight estimation, incomplete data, region removal, and feature-weight perturbation. For analyses that compare cluster assignments, agreement with the primary clustering solution is assessed using the Adjusted Rand Index (ARI) and Adjusted Mutual Information (AMI). These metrics are appropriate for this setting because they compare two partitions of the same spatial units while adjusting for agreement expected by chance. ARI measures pairwise agreement in cluster co-assignment, whereas AMI provides an information-theoretic measure of shared clustering structure. Prior clustering literature suggests that ARI values greater than 0.65 indicate moderate agreement between clustering solutions (Steinley, 2004). Therefore, ARI values near this threshold, together with consistent AMI values, are interpreted as evidence that the overall clustering structure is reasonably preserved, while recognizing that some regional reassignment may still occur.

### 4.1. Weighting scheme

To assess the influence of outcome-informed feature weighting, we compare the weighted hierarchical clustering solution with an otherwise unweighted solution. The two solutions show moderate-to-strong agreement (ARI = 0.695; AMI = 0.645). These results indicate that the overall clustering structure remains reasonably stable after removing the weighting scheme, although some regional assignments are affected by weighting. The visual comparison in Figure S3 further shows that the weighted and unweighted solutions produce broadly similar spatial clustering patterns.

### 4.2. Weight stability

To evaluate the stability of outcome-informed feature weights, we repeat the weighting procedure 100 times using independently sampled weighting subsets. For each run, feature weights are recomputed and compared with the default weights used in the primary analysis. Across the 100 repeated sampling runs, the outcome-informed weights are highly stable, with an average relative variation of 2.8% across features. The sampled weights are also highly consistent with the default weights in both magnitude and ranking, with mean Pearson and Spearman correlations of 0.998 and 0.984, respectively. These results indicate that the weighting procedure is not strongly driven by a particular random weighting subset and that the relative importance of structural features remains highly consistent across repeated samples.

### 4.3. Data completeness

In the clustering procedure, region-level structural characteristics are represented by mean values aggregated over nine years. As such, the resulting summaries may be influenced by incomplete observations. To assess the sensitivity of the clustering solution to data completeness, we conduct a perturbation analysis by randomly excluding a proportion of samples prior to the clustering step. Specifically, for each region, 20% of quarterly observations are randomly removed, after which the structural feature means are recalculated. The clustering procedure is then repeated 100 times using the perturbed data, and the resulting solutions are compared with the original clustering using ARI and AMI. As illustrated in Figure S4 in the Supplementary Material, the mean agreement between the original clustering and the perturbed solutions is adequate (ARI = 0.625; AMI = 0.658). The mean ARI is close to the moderate-agreement threshold, and the corresponding AMI value is consistent with this interpretation. These results indicate that while some reassignment occurs under data incompleteness, the overall structural configuration remains preserved.

### 4.4. Region inclusion

The clustering solution may also depend on which regions are included in the analysis. In response, we randomly remove 10% of the regions and repeat the clustering procedure using the same configuration as described above. The agreement is comparable to that derived under data completeness perturbation (ARI = 0.640; AMI = 0.665), as illustrated in Figure S5 in the Supplementary Material. These values again approach the moderate-agreement range, suggesting that the main partition is broadly preserved even when a subset of regions is removed. The comparison indicates that the clustering solution is somewhat sensitive to regional inclusion, while the overall clustering structure remains stable.

### 4.5. Feature weighting

The population clustering applies an outcome-informed feature weighting strategy to account for the fact that structural characteristics may contribute unequally to the health outcome. Rather than treating all features as uniformly influential, the weighting scheme assigns differential emphasis according to their estimated relevance, thereby incorporating heterogeneity in structural influence into the clustering process. To evaluate the sensitivity of this weighting specification, we introduce random variations of ±5% to all feature weights. The clustering procedure otherwise follows the same experimental settings as described above. As illustrated in Figure S6 in the Supplementary Material, the mean agreement under weight variation is still moderate and stable (ARI = 0.638; AMI = 0.685). The degree of agreement indicates that small perturbations to feature weights do not materially affect the resulting cluster membership.

Across the sensitivity analyses, the similar agreement levels and distribution patterns suggest a coherent robustness profile of the clustering solution. The comparison between weighted and unweighted solutions shows that the main spatial structure is broadly preserved even without outcome-informed weighting, and the repeated weight-estimation analysis indicates that the feature weights themselves are highly stable. For the cluster-assignment perturbation analyses, the mean ARI values are close to the moderate-agreement threshold across data-completeness perturbation, region-removal sensitivity, and feature-weight perturbation, while the corresponding AMI values provide consistent information-theoretic support for the same pattern of preserved agreement. While localized reassignment occurs under varying configurations, the overall partition of regions stays stable, and no single perturbation scenario leads to pronounced structural disruption.

## 5. Discussion

In this study, we find that group-specific modeling improves predictive performance relative to statewide pooled modeling. This improvement may reflect heterogeneous population vulnerability in exposure-related asthma responses rather than a uniform statewide exposure–asthma association. In other words, regions with similar pollutant and weather conditions may still show different asthma hospitalization patterns across population groups. This interpretation is informed by the systematic divergence between statewide pooled modeling and group-specific modeling. At the overall level, group-specific modeling consistently achieved superior predictive performance relative to statewide pooled modeling across repeated iterations. Importantly, the magnitude and direction of performance changes when comparing the two strategies varied across groups, with certain population types exhibiting marked improvements under group-specific modeling while others showed minimal or mixed changes. This structured divergence suggests that representing exposure–asthma patterns through a single scalar value or simple aggregation of structural variables may overlook important vulnerability patterns that become more apparent when examined by population type. Specifically, populations are situated within different baseline health statuses, socioeconomic conditions, and access-to-care environments, factors that may be associated with asthma exacerbation severity and hospitalization risk. When such heterogeneous populations are examined within a single analytical framework, the resulting estimate reflects only an average exposure–asthma association. In contrast, stratified analysis enables these associations to be examined more clearly across groups. Accordingly, these findings underscore the importance of vulnerability stratification when evaluating exposure–asthma patterns in heterogeneous populations.

Within this framework, population clustering serves to operationalize vulnerability stratification by identifying structurally coherent population types. Rather than serving merely as descriptive categories, these population types are directly incorporated into exposure–asthma modeling, enabling the evaluation of heterogeneous predictive patterns across vulnerability profiles. Although related strategies have been explored in selected areas of environmental health research—for example, Ljungman et al. (2016) constructed clusters based on multi-pollutant mixtures to examine associations with microvascular function—such approaches remain uncommon in asthma research. In analyses of asthma in relation to environmental exposures, structural characteristics often account for substantial variation, whereas the predictive contributions of pollutant and meteorological variables may appear comparatively subtle. When estimated within pooled models, dominant structural gradients can obscure heterogeneous exposure–asthma patterns. Vulnerability stratification, by contrast, allows exposure–asthma patterns to be examined across population vulnerability contexts. In this design, downstream modeling is not introduced to enhance prediction accuracy, but to assess whether population types meaningfully differentiate exposure–asthma predictive patterns. Clustering is conducted as an independent step and is not iteratively adjusted based on downstream model performance. This separation positions downstream modeling as an evaluative component, so that performance divergence can serve as evidence that group-specific models better capture predictive structure within the identified population types, rather than reflecting a direct consequence of optimizing the clustering solution. The improved predictive fit also aligns with the presence of heterogeneity across population vulnerability contexts. However, it should not be interpreted as direct estimation or comparison of cluster-specific exposure–response parameters or functions.

As an extension of the methodology, the group-specific modeling results for five population types may reflect differential susceptibility or contextual factors associated with exposure-related asthma hospitalization. The Upland–Plateau group exhibits an asthma hospitalization distribution closely aligned with the statewide pattern, and stratified modeling yields results similar to pooled analysis. This alignment suggests that exposure–asthma predictive patterns in this cluster follow the broader statewide configuration, with limited group-specific divergence. Within this group, exposure variables account for a measurable portion of the variation in hospitalization, indicating the presence of a detectable exposure–asthma predictive signal. The Lowland–Valley group demonstrates a clear departure from the statewide configuration. Under pooled modeling, exposure variables exhibit little to no explanatory power. For group-specific modeling, however, positive explanatory performance emerges, suggesting that some predictive information obscured in the statewide model may be partially captured when this subgroup is modeled separately. Notably, the Lowland–Valley constitutes the second largest group, accounting for more than one quarter of all regions. Thus, the performance gains from group-specific modeling are unlikely to stem solely from insufficient representation in pooled modeling; rather, they may correspond to a distinct vulnerability configuration specific to this group. Similarly, the Peri-Urban group is distinct from the broader statewide configuration. Under group-specific modeling, explanatory power improves, and exposure variables show stronger predictive relevance. Yet, the high variability in predictive performance suggests limited stability. One possible explanation is that this instability is related to the structurally mixed urban context of this group, although this interpretation remains tentative. Unlike other groups, the Affluent Suburb group exhibits the lowest asthma hospitalization burden, with clear misalignment under statewide pooled modeling. While group-specific modeling corrects this misalignment, overall explanatory performance remains minimal, with exposure variables showing limited predictive relevance. This interpretation is compatible with the relatively advantaged socioeconomic and environmental profile of this group, although it remains exploratory. Nonetheless, this still represents a form of group-specific divergence, with a notably weaker exposure–asthma pattern than the statewide one. The Urban Core group carries the greatest asthma burden with inconsistent shifts in model performance among different evaluation metrics. The multi-peaked age distribution suggests the presence of subpopulations with differing vulnerabilities, which may represent one source of instability in model performance. Spatially, these regions also show structural features consistent with dense urban disadvantage, including high population density and elevated low-income composition. Given the substantial asthma burden, socioeconomic disadvantage, and heterogeneous subpopulations, the Urban Core warrants a more nuanced examination of exposure–asthma patterns. Broadly, a gradient emerges from non-urban landscapes to urban areas, with asthma burden tending to increase and exposure-related model behavior appearing more complex. This trend is consistent with prior research conducted in other regions of the United States.

The breadth and integration of data sources enhance the methodological robustness of this study. Spanning multiple structural and environmental domains over nine years across the state, this populationlevel approach strengthens analytical stability and provides a comprehensive depiction of population heterogeneity. Nevertheless, several limitations should be acknowledged to clarify the scope of interpretation. First, the analysis is conducted at the regional level. Although the aggregation of ZCTAs into larger regions was implemented to ensure sufficient population size and improve estimation stability, regional-level findings may not directly reflect individual-level vulnerability, and finer-scale heterogeneity within regions may be masked. Relatedly, the resulting population types may be influenced by the selected regionalization procedure, population threshold, adjacency definition, and spatial unit. Alternative aggregation schemes or regionalization strategies could therefore produce somewhat different vulnerability profiles. Second, the analysis may be affected by residual contextual factors that are not fully captured in the available datasets. The absence of individual-level covariates limits the ability to account for within-region differences. Unmeasured factors such as healthcare access, smoking prevalence, indoor exposures, housing conditions, insurance coverage, and healthcare-seeking behavior may also contribute to asthma hospitalization patterns. In addition, the uneven distribution of regions across population types, particularly the relatively smaller number of Urban Core regions, may reduce precision in group-specific modeling, and related comparisons should therefore be interpreted with appropriate caution. Third, the clustering framework involves methodological choices that should be considered when interpreting the results. Although the selection of five clusters is supported by internal validity metrics and substantive interpretability, it still involves judgment in balancing parsimony, separation, and structural differentiation. Sensitivity analyses indicate that the broad clustering structure is preserved, but the moderate ARI values also suggest non-trivial regional reassignment under perturbation. Similarly, although outcome-informed feature weighting is estimated using an independent subset and evaluated through sensitivity analyses, it still introduces asthma outcome information into the clustering process.

Future work could compare alternative weighting strategies, externally defined vulnerability indices, and additional clustering specifications to further evaluate the robustness of population stratification. Beyond Pennsylvania, the framework may provide a basis for stratifying populations in other geographic settings and related respiratory outcomes, but such applications would require recalibration and external validation because the identified population types are derived from Pennsylvania-specific socioeconomic, environmental, geographic, and healthcare contexts. A complementary direction is to examine potential group-specific mechanisms that may underlie exposure–asthma associations by investigating variable importance patterns, nonlinear model structures, and additional contextual factors. Building on the present study, if exposure–asthma relationships within distinct populations can be further identified, these insights may help inform contextspecific surveillance priorities, warning thresholds, and preventive strategies.

## 6. Conclusion

This study proposes a data-driven framework for evaluating asthma burden through population clustering and group-specific exposure–outcome modeling. Using clustering to define population strata and treating downstream model performance as an evaluative component, the framework assesses whether group-specific models better capture predictive structure within the identified population types. Applied to statewide data from Pennsylvania (2011–2019), the stratified modeling approach improves predictive performance relative to statewide pooled modeling, a finding consistent with the presence of heterogeneity across population vulnerability contexts. The framework may be adaptable to broader multi-state settings and related respiratory conditions, but such applications would require recalibration and external validation.

## Supplementary Material

1

## Figures and Tables

**Fig. 1.**
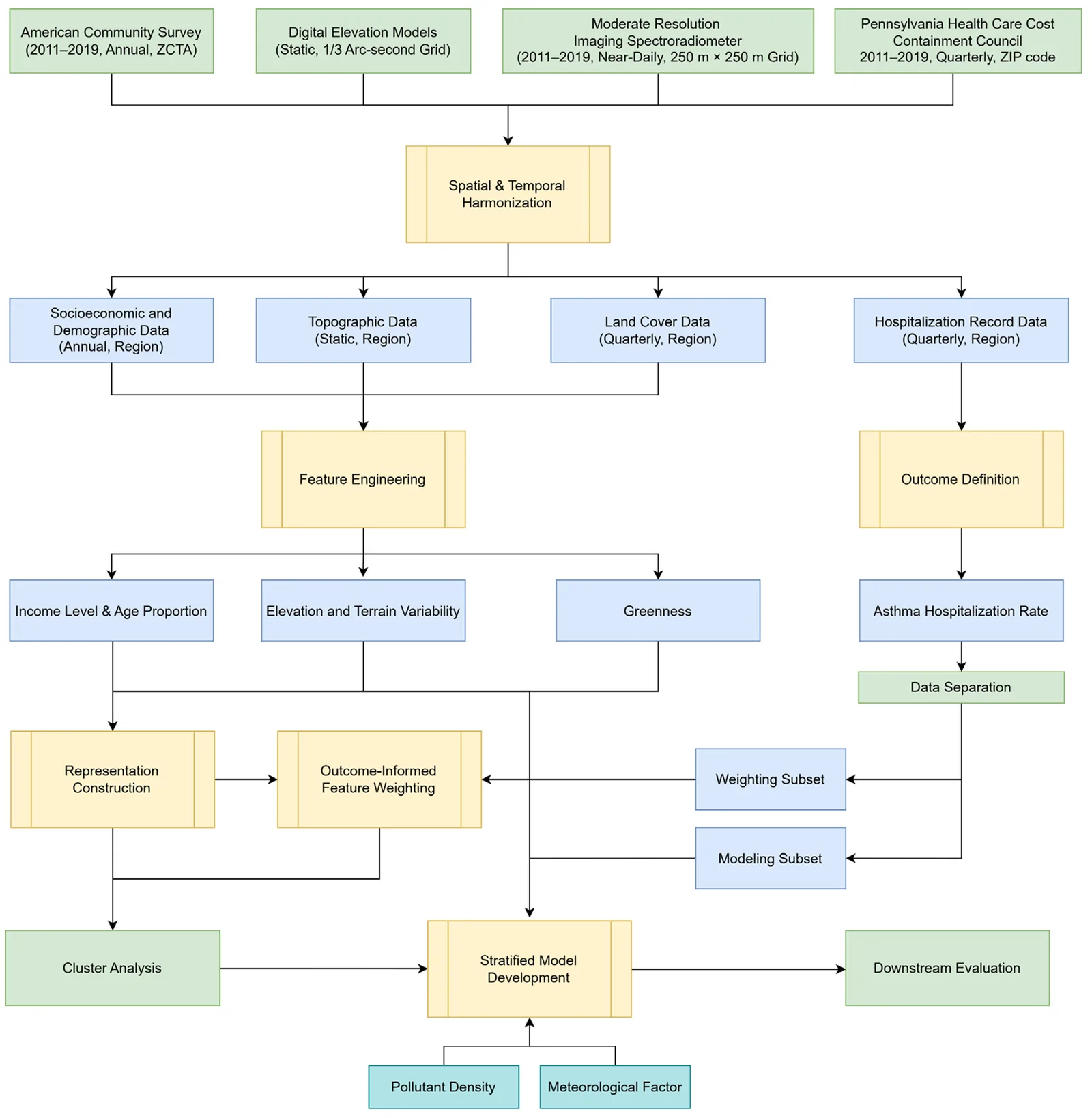
Overview of the integrated data workflow.

**Fig. 2.**
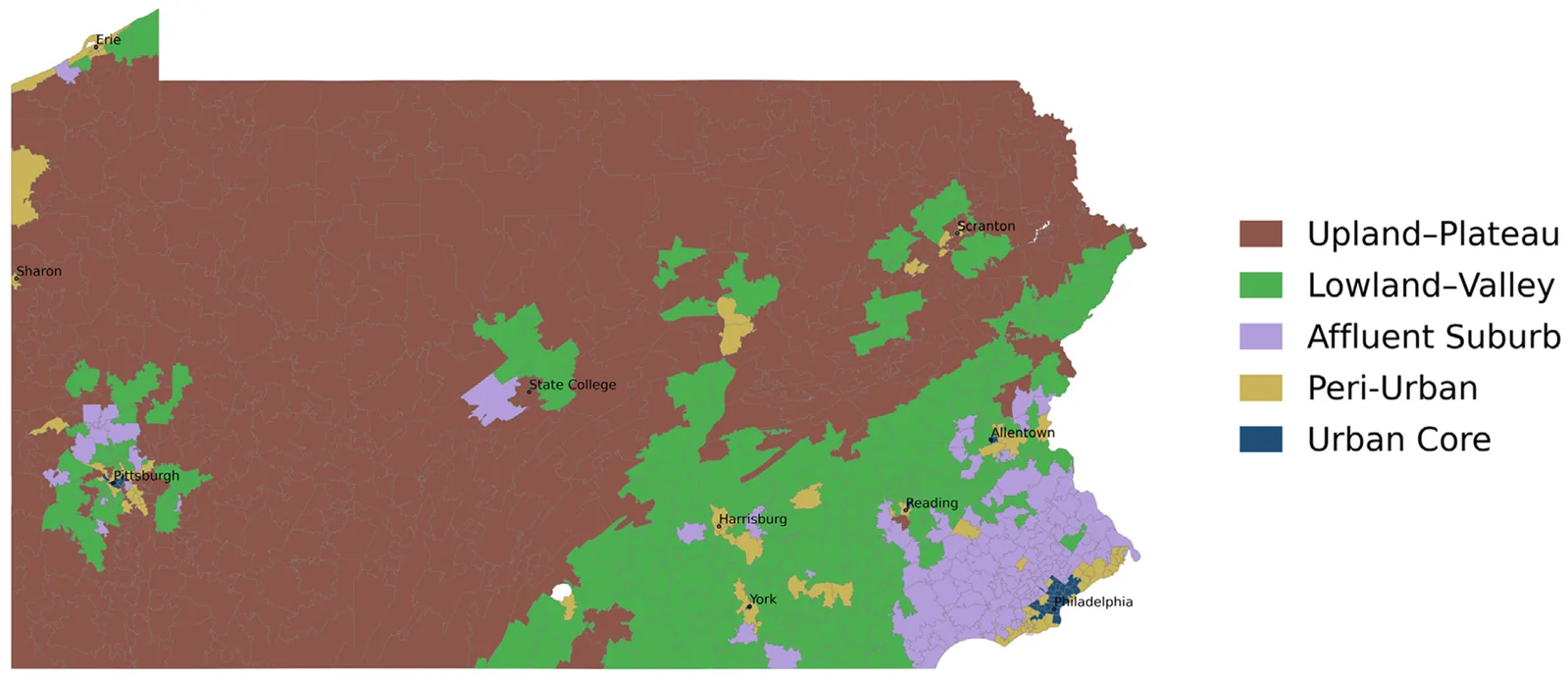
Geographic distribution of the five region clusters across Pennsylvania. Each color denotes a distinct cluster identified by the selected clustering solution (*k* = 5), illustrating the spatial coverage and relative distribution of the Upland–Plateau, Lowland–Valley, Affluent Suburb, Peri-Urban, and Urban Core groups. Map lines delineate study areas and do not necessarily depict accepted national boundaries.

**Fig. 3.**
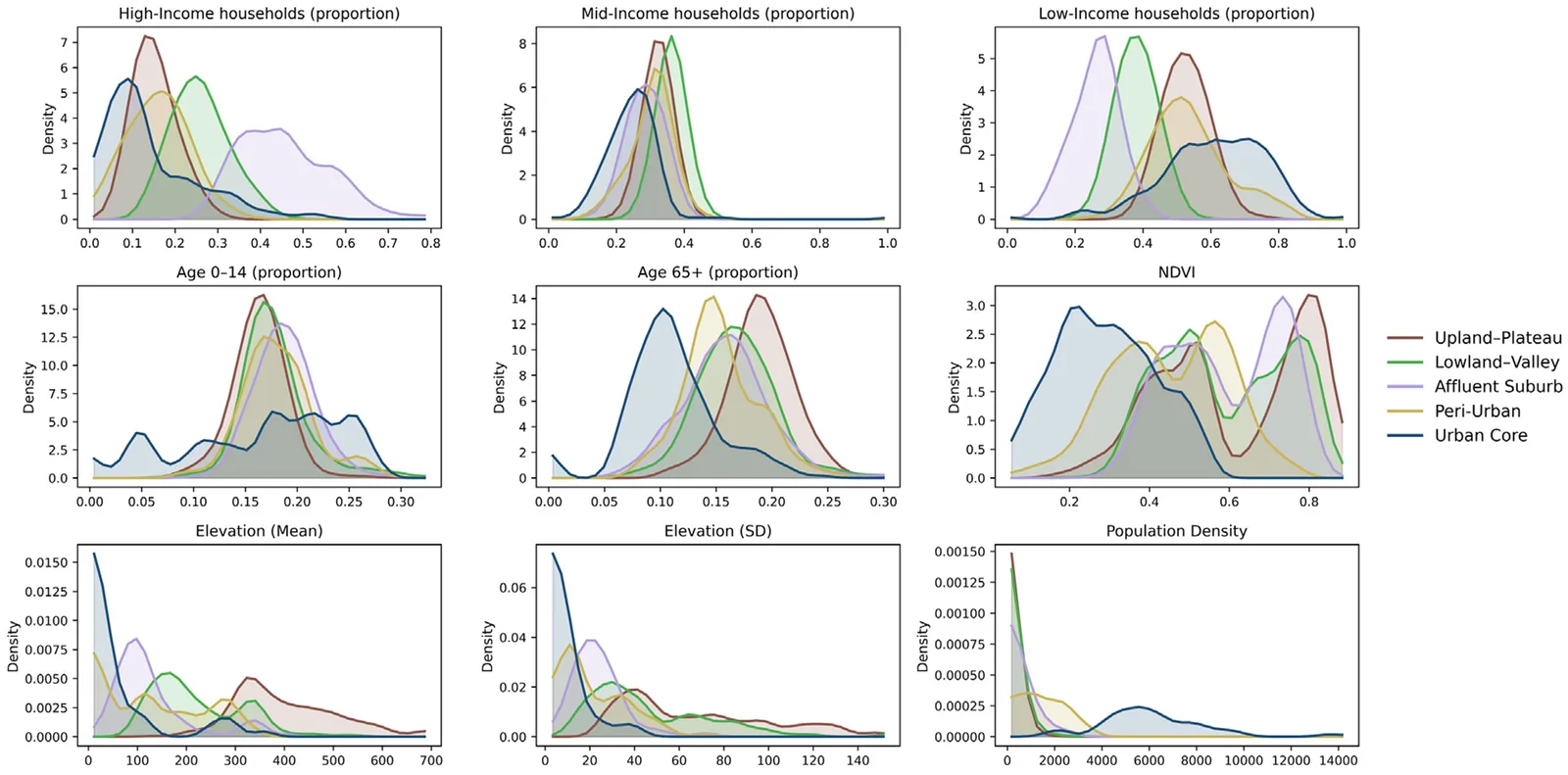
Smoothed distributions of structural variables across the identified groups. Each curve is constructed from region-level feature values, where each region is represented by its mean value within that spatial unit (i.e., one observation per region). For each feature, group-specific empirical densities are estimated using normalized histograms (with a common set of bin edges shared across clusters within the same panel) and then smoothed by applying a one-dimensional Gaussian filter to the histogram densities, yielding continuous curves for visual comparison.

**Fig. 4.**
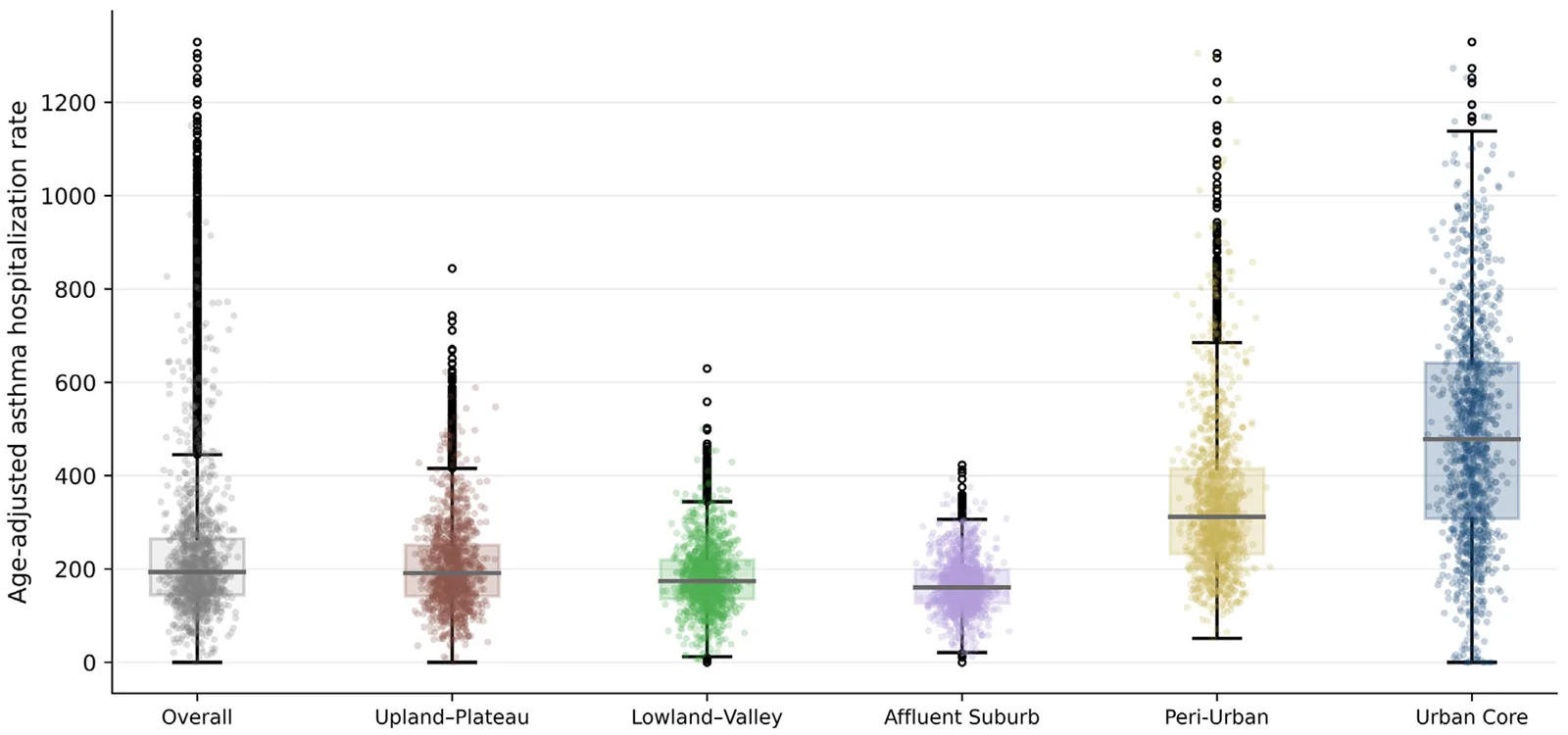
Distribution of the age-adjusted asthma hospitalization rate. The *Overall* group represents the statewide distribution, serving as a reference baseline. For each group, the box indicates the interquartile range (IQR) with the median shown as a dark gray line; whiskers and outliers follow standard boxplot conventions. Jittered points are overlaid to visualize dispersion and the density of observations.

**Table 1:** Factor loading patterns for income and age composition variables. The loadings were used to guide the aggregation of original ACS categories into interpretable structural variables for clustering.

(a) Income categories			
Feature	Factor 1	Factor 2	Factor 3

<$10k	0.057	0.063	0.977
$10–15k	0.272	0.015	0.951
$15–25k	0.466	0.078	0.869
$25–35k	0.578	0.138	0.791
$35–50k	0.695	0.215	0.674
$50–75k	0.783	0.345	0.506
$75–100k	0.791	0.499	0.334
$100–150k	0.641	0.737	0.156
$150–200k	0.376	0.912	0.053
$200k+	0.057	0.981	0.023

(b) Age categories			
Feature	Factor 1	Factor 2	Factor 3

<5	0.829	0.378	0.368
5–9	0.824	0.349	0.417
10–14	0.819	0.330	0.427
15–19	0.436	0.783	0.319
20–24	0.281	0.912	0.236
25–34	0.669	0.476	0.402
35–44	0.764	0.351	0.522
45–54	0.713	0.308	0.608
55–59	0.648	0.298	0.681
60–64	0.594	0.293	0.729
65–74	0.521	0.275	0.792
75–84	0.409	0.253	0.865
85+	0.281	0.268	0.890

**Table 2:** Internal validity metrics for candidate clustering resolutions. Silhouette scores quantify average cluster separation, explained variability represents the proportion of total variance explained by the clustering solution, and incremental gain denotes the additional explained variability obtained by increasing the number of clusters from the previous resolution.

*k*	Silhouette score	Explained variability	Incremental gain
3	0.310	0.418	–
4	0.226	0.513	0.095
5	0.224	0.589	0.076
6	0.224	0.614	0.025
7	0.197	0.636	0.022
8	0.145	0.654	0.018
9	0.148	0.672	0.018
10	0.144	0.688	0.016

**Table 3:** Descriptive statistics of socioeconomic, demographic, and landscape features across the identified groups.

Variable	Upland–Plateau	Lowland–Valley	Affluent Suburb	Peri-Urban	Urban Core
High-Income households (proportion)	0.152 (0.114, 0.184)	0.258 (0.208, 0.301)	0.455 (0.373, 0.530)	0.162 (0.111, 0.210)	0.146 (0.068, 0.208)
Mid-Income households (proportion)	0.322 (0.299, 0.347)	0.362 (0.338, 0.388)	0.285 (0.248, 0.324)	0.303 (0.274, 0.340)	0.244 (0.202, 0.286)
Low-Income households (proportion)	0.526 (0.478, 0.573)	0.380 (0.335, 0.421)	0.260 (0.214, 0.304)	0.535 (0.454, 0.598)	0.609 (0.517, 0.720)
Age 0–14 (proportion)	0.163 (0.149, 0.179)	0.178 (0.159, 0.191)	0.187 (0.168, 0.205)	0.181 (0.159, 0.200)	0.168 (0.113, 0.227)
Age 65+ (proportion)	0.190 (0.173, 0.209)	0.167 (0.145, 0.189)	0.158 (0.134, 0.180)	0.154 (0.135, 0.174)	0.113 (0.088, 0.132)
NDVI	0.611 (0.453, 0.787)	0.593 (0.461, 0.742)	0.593 (0.473, 0.723)	0.453 (0.342, 0.569)	0.292 (0.198, 0.385)
Elevation (Mean)	399.038 (324.259, 468.994)	221.392 (150.106, 302.684)	128.639 (81.001, 136.927)	141.628 (31.690, 242.592)	69.711 (15.535, 78.070)
Elevation (SD)	67.069 (39.551, 90.732)	46.108 (25.901, 64.073)	24.005 (16.484, 29.547)	21.884 (10.288, 33.417)	10.816 (5.126, 12.898)
Population Density	138.735 (23.889, 145.349)	283.103 (71.476, 306.492)	679.065 (252.083, 871.815)	1545.924 (731.502, 2269.952)	6048.720 (4747.040, 7299.080)

*Notes:* Values are reported as medians; interquartile ranges are shown in parentheses.

**Table 4:** Predictive performance comparison between the statewide and group-specific models across 100 random splits.

Cluster	R^2^			RMSE		
	Statewide	Group-specific	*ΔR* ^2^	Statewide	Group-specific	*Δ*RMSE
Overall	0.437 ± 0.107	0.501 ± 0.091	0.064	101.59 ± 7.88	97.19 ± 8.14	4.39
Upland–Plateau	0.255 ± 0.090	0.255 ± 0.098	−0.001	77.65 ± 5.10	77.32 ± 5.25	0.33
Lowland–Valley	−0.110 ± 0.167	0.147 ± 0.051	0.257	71.61 ± 4.97	62.45 ± 3.62	9.16
Affluent Suburb	−1.411 ± 1.275	0.025 ± 0.076	1.436	84.28 ± 20.25	55.31 ± 3.30	28.97
Peri-Urban	0.065 ± 0.269	0.151 ± 0.224	0.086	149.13 ± 27.46	145.09 ± 28.50	4.04
Urban Core	−0.045 ± 0.680	0.069 ± 0.360	0.114	210.14 ± 39.85	216.88 ± 35.36	−6.74

*Notes:* Values are reported as mean ± standard deviation. *ΔR*^2^ is computed as group-specific–statewide, and *Δ*RMSE as statewide–group-specific, such that positive values indicate improvement under the stratified framework.

## Data Availability

Publicly available datasets were analyzed in this study. Socioeconomic data were obtained from the American Community Survey (ACS), topographic data from the National Aeronautics and Space Administration (NASA) Digital Elevation Models (DEM), land cover data from the Moderate Resolution Imaging Spectroradiometer (MODIS), pollutant data from the Community Multiscale Air Quality (CMAQ) modeling system, and meteorological data from the Gridded Surface Meteorological Dataset (gridMET). These datasets are available from their respective public repositories. Hospitalization records were obtained from the Pennsylvania Health Care Cost Containment Council (PHC4) and are subject to data use agreements and privacy restrictions. These data are not publicly available.

## Appendix A. Supplementary data

Supplementary material related to this article can be found online at https://doi.org/10.1016/j.envadv.2026.100735.
