# Supplementary material for: Hierarchical clustering for disentangling population vulnerability to asthma
Source: Environ Adv. Author manuscript; Available in PMC 2026 Oct 14. (PMC13660370; doi:10.1016/j.envadv.2026.100735)
Supplement: Supplementary file 1 [file NIHMS2209979-supplement-1.pdf]

Supplementary Material

This supplementary material provides additional figures supporting the main analyses presented in the manuscript. Figure S1 illustrates the final spatial units (regions) after ZCTA aggregation. Figure S2 presents internal validity diagnostics for selecting the number of clusters. Figure S3 compares the weighted and unweighted hierarchical clustering solutions. Figures S4–S6 summarize robustness analyses under data perturbation and feature-weight variation.

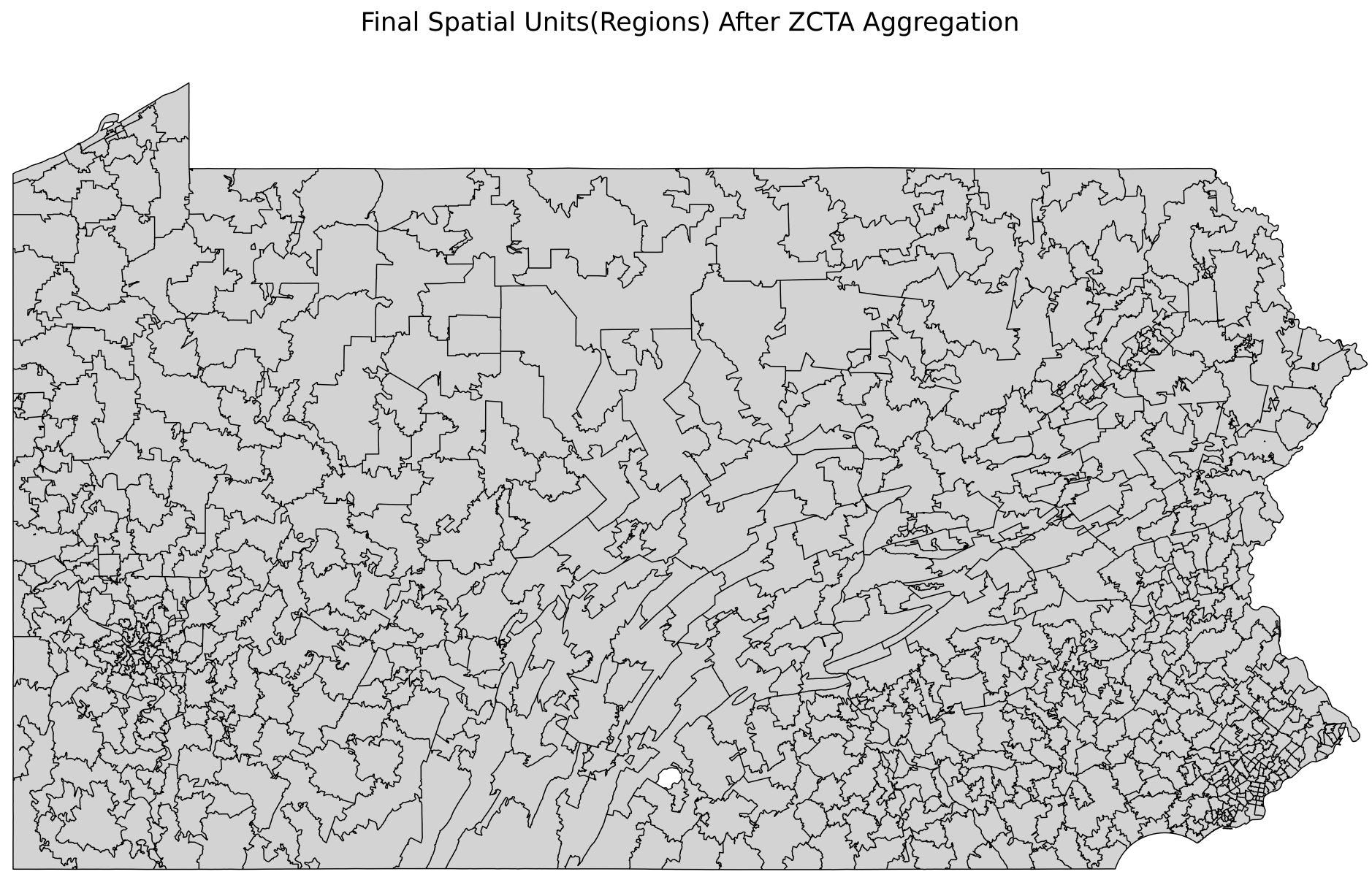

Figure S1: Final spatial units (regions) after ZCTA aggregation across Pennsylvania. A total of 745 regions are constructed, each comprising one or more ZCTAs. All regions exceed a total population of 5,000 residents in every study year and serve as the fundamental spatial units for subsequent analyses. Map lines delineate study areas and do not necessarily depict accepted national boundaries.

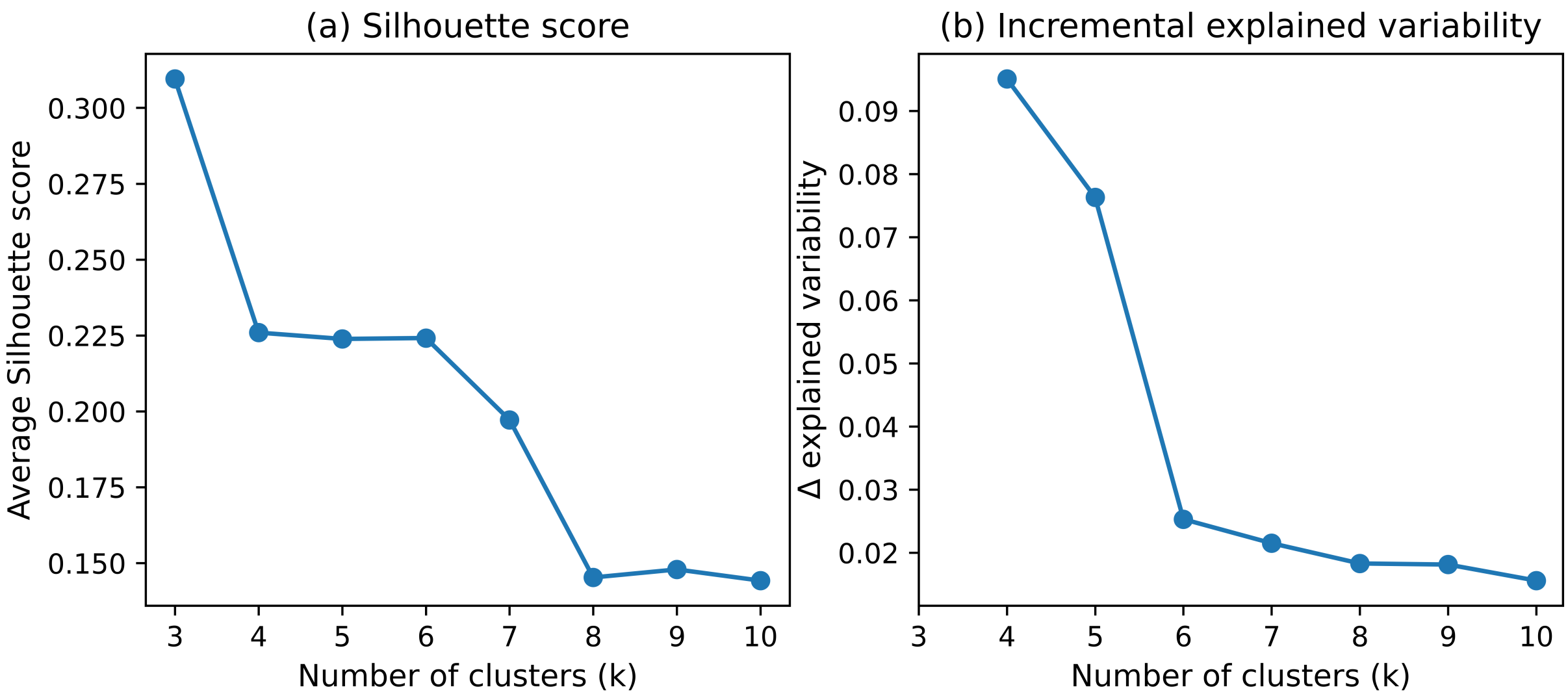

Figure S2: Internal validity criteria for selecting the number of clusters. (**a**) Average Silhouette score as a function of the number of clusters k, attaining its maximum at $k = 3$ and remaining relatively stable from $k = 4$ to 6. (**b**) Incremental explained variability as a function of k, showing a marked reduction in marginal gain at $k = 6$.

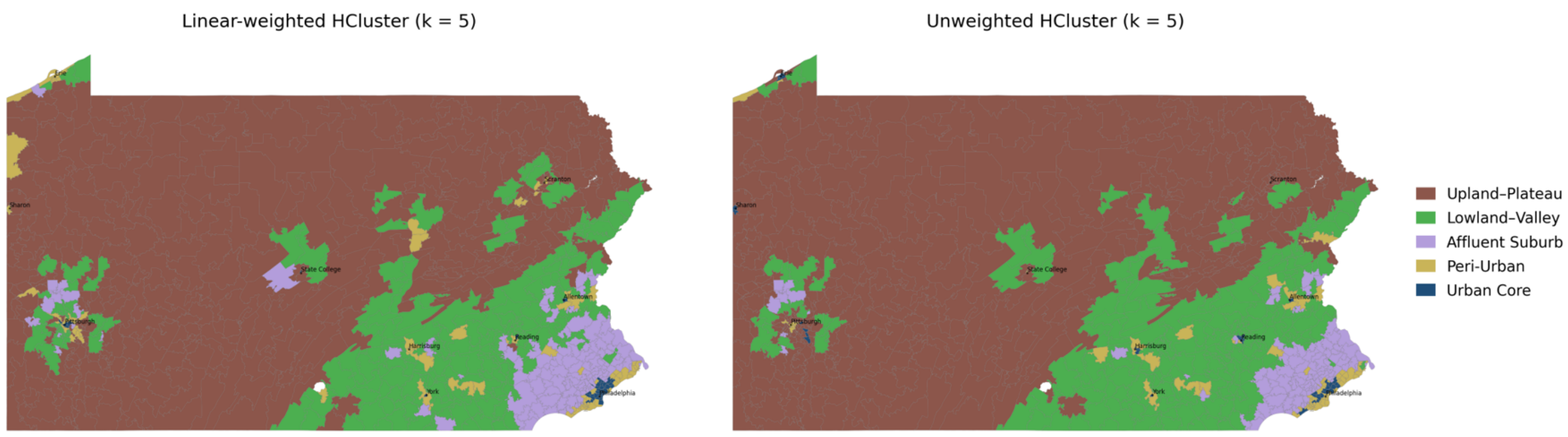

Figure S3: Comparison of the weighted and unweighted hierarchical clustering solutions at $k = 5$. The left panel shows the linear-weighted hierarchical clustering result, and the right panel shows the corresponding unweighted solution.

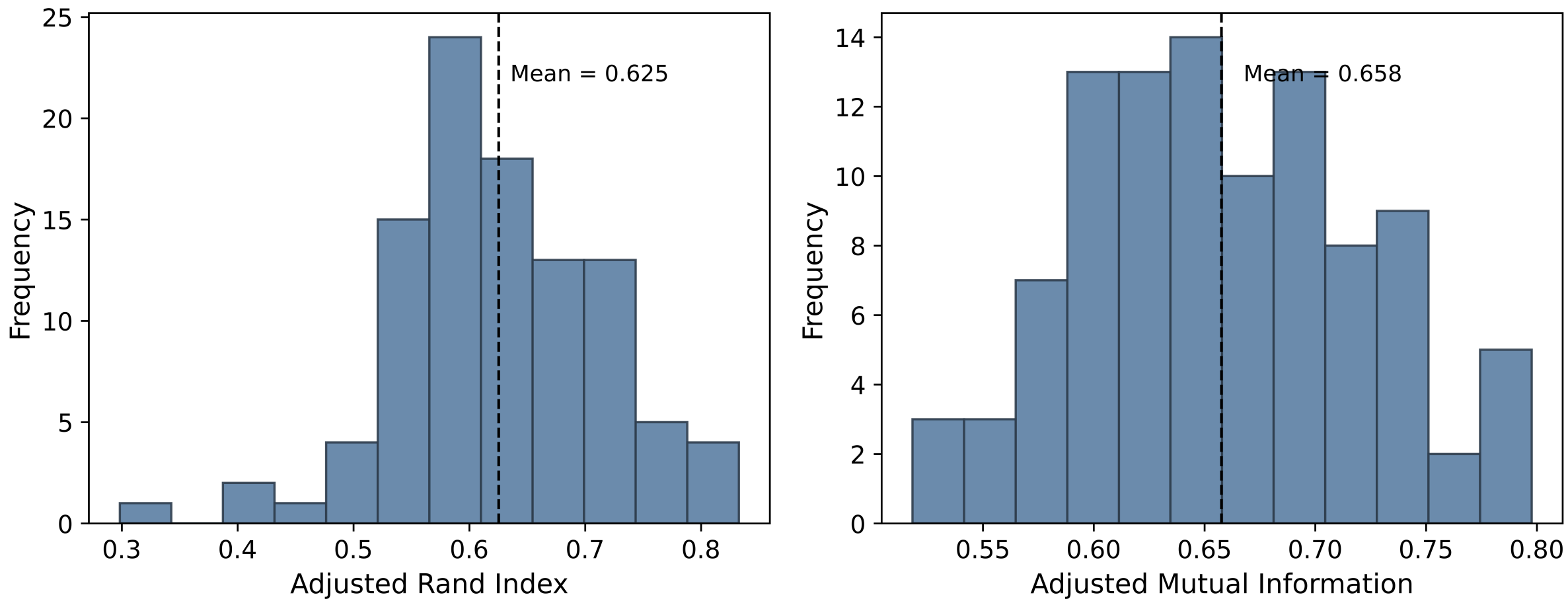

Figure S4: Distribution of ARI and AMI under 20% data removal across 100 perturbation runs. The dashed lines indicate mean agreement with the original clustering.

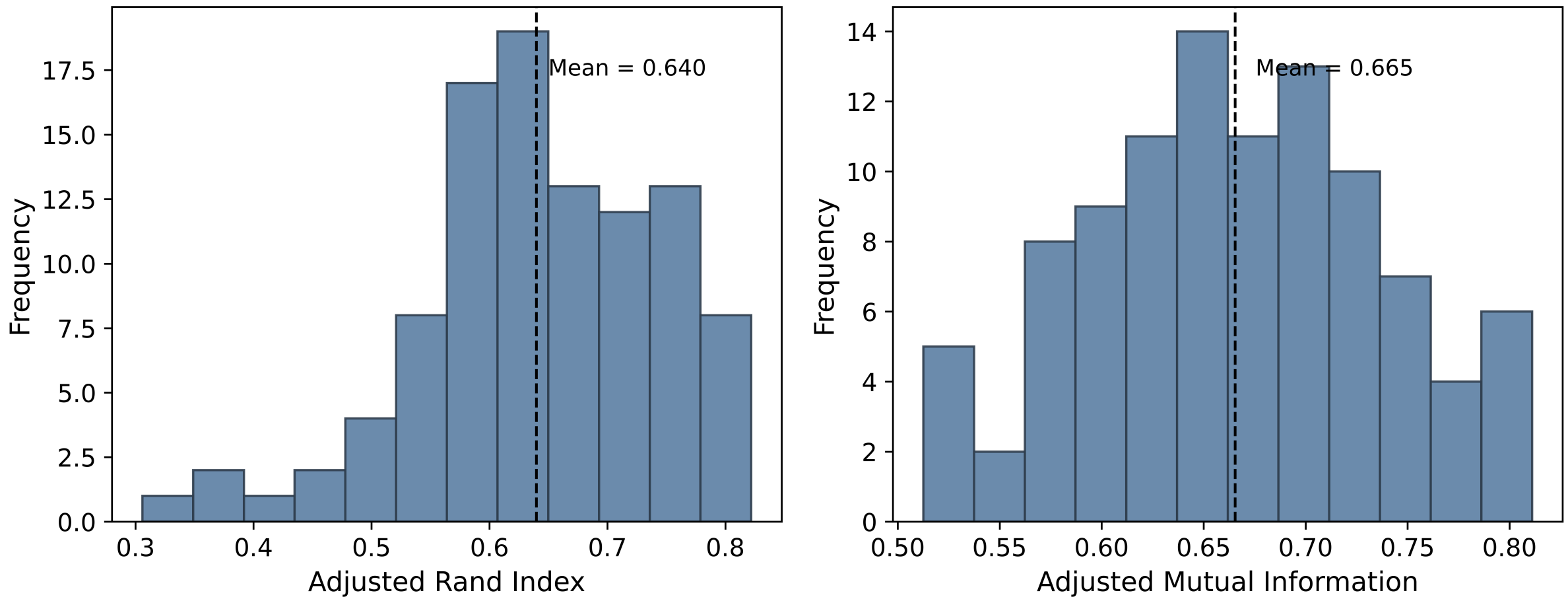

Figure S5: Distribution of ARI and AMI under 10% regional removal across 100 perturbation runs. The dashed lines indicate mean agreement with the original clustering.

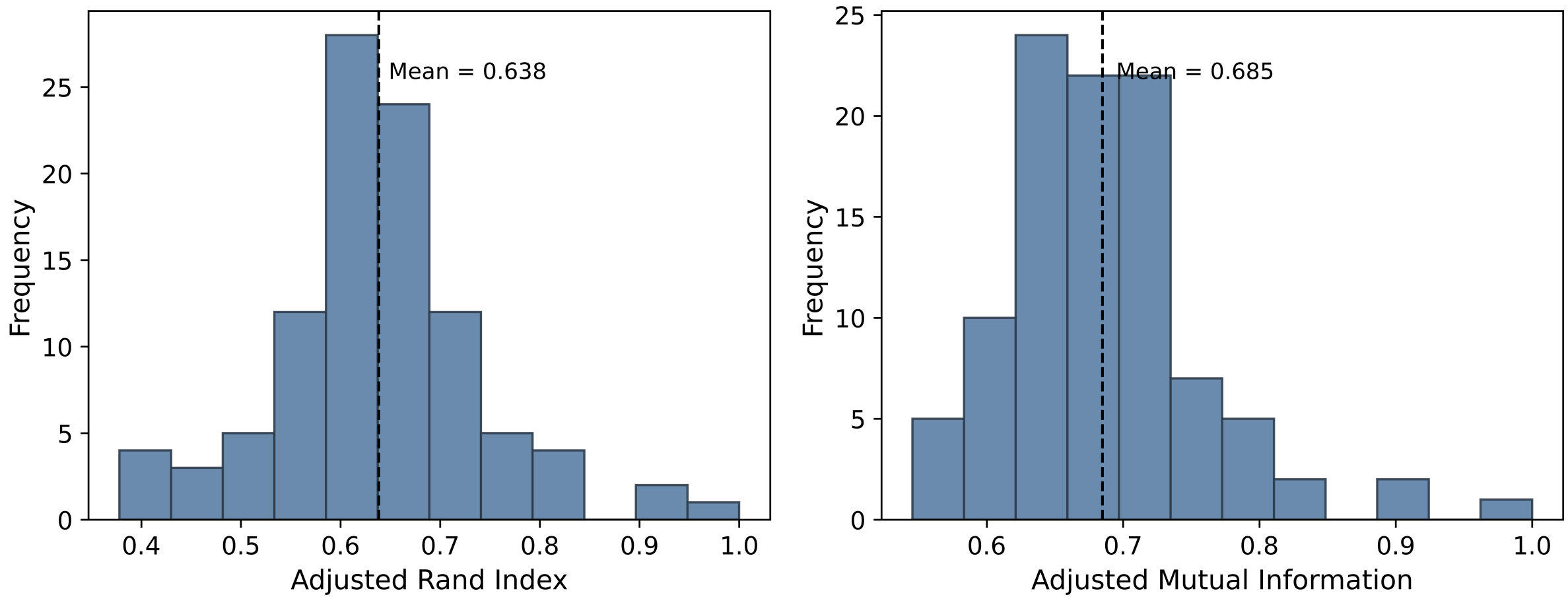

Figure S6: Distribution of ARI and AMI under random feature-weight perturbations of ±5% across 100 iterations. The dashed lines indicate the mean agreement with the original clustering.
